# Regulatory T‐cells in helminth infection: induction, function and therapeutic potential

**DOI:** 10.1111/imm.13190

**Published:** 2020-04-19

**Authors:** Madeleine P. J. White, Caitlin M. McManus, Rick M. Maizels

**Affiliations:** ^1^ Wellcome Centre for Integrative Parasitology Institute of Infection, Immunity and Inflammation University of Glasgow Glasgow UK

**Keywords:** immune regulation, immunomodulators, inflammation, therapy

## Abstract

Helminth parasites infect an alarmingly large proportion of the world's population, primarily within tropical regions, and their ability to down‐modulate host immunity is key to their persistence. Helminths have developed multiple mechanisms that induce a state of hyporesponsiveness or immune suppression within the host; of particular interest are mechanisms that drive the induction of regulatory T‐cells (Tregs). Helminths actively induce Tregs either directly by secreting factors, such as the TGF‐β mimic *Hp‐*TGM, or indirectly by interacting with bystander cell types such as dendritic cells and macrophages that then induce Tregs. Expansion of Tregs not only enhances parasite survival but, in cases such as filarial infection, Tregs also play a role in preventing parasite‐associated pathologies. Furthermore, Tregs generated during helminth infection have been associated with suppression of bystander immunopathologies in a range of inflammatory conditions such as allergy and autoimmune disease. In this review, we discuss evidence from natural and experimental infections that point to the pathways and molecules involved in helminth Treg induction, and postulate how parasite‐derived molecules and/or Tregs might be applied as anti‐inflammatory therapies in the future.

## Graphical abstract

Helminth parasites successfully colonize many niches in the body, and potently manipulate the regulatory T‐cell network in each environment. In particular, qualitative changes to activation markers and increased suppressive function accompany a numerical expansion in both natural and induced Tregs that prolong infection and dampen responses to bystander antigens such as allergens.

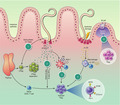

**Abbreviations**
Ab: antibody
Ag: antigen
ANA: anti‐nuclear antibodies
Breg: regulatory B‐cells
CTLA‐4: cytotoxic T‐lymphocyte‐associated protein 4
DC: dendritic cell
ES: excretory/secretory product
Foxp3: forkhead box P3
GITR: glucocorticoid‐induced tumour necrosis factor receptor
HDM: house dust mite
HES: *H. polygyrus* excretory‐secretory product
*Hp‐*TGM: *H. polygyrus* TGM (TGF‐β mimic)
IBD: inflammatory bowel disease
ICOS: inducible T‐cell co‐stimulator
IDO‐1: indolamine 2,3‐dioxygenase
IFN‐γ: interferon gamma
iTregs: induced Tregs
MS: multiple sclerosis
NOD: non‐obese diabetic
nTregs: natural Tregs
OVA: ovalbumin
PD‐1: programmed cell death protein 1
PD‐L1: programmed death‐ligand 1
PP: Peyer's patch
pTreg: peripheral Tregs
RA: retinoic acid
RORγt: RAR‐related orphan receptor gamma
SEA: soluble egg antigens
TGF‐β: transforming growth factor beta
TIGIT: T‐cell immunoreceptor with Ig and ITIM domains
Tr1: type 1 regulatory cells
TSO: *Trichuris suis* ova

## Introduction

Helminth worm parasite infections currently afflict one‐quarter of the world's population,^1^, ^2^ the majority of whom are located in resource‐poor tropical countries. However, before sanitation improvements and industrialization became more widespread in the last century, the prevalence of helminths was likely to be high across the globe. Alongside the disappearance of helminth infections from the higher‐income countries, there have however been sharp rises in a suite of inflammatory autoimmune and allergic disorders. One possibility, suggested by the ‘hygiene hypothesis’ and more recently the ‘old friends hypothesis’ is that helminths are one of the key environmental influences, along with members of the microbial world, that dampen immune reactivity to innocuous bystander antigens.^3^, ^4^, ^5^, ^6^ While the relative importance of each environmental factor in restraining inflammatory processes has yet to be established, the ability of many helminth parasites to downregulate the host immune system suggests that they may play a major role in regulating immune disorders in humans.^7^

Immune regulation by helminths acts at many levels to interfere with innate antigen sensitization, induction of adaptive immunity, and mobilization of effector mechanisms.^7^ One of the most prominent pathways for parasite immunomodulation is through regulatory T‐cells (Tregs). Tregs are classified by their expression of the transcription factor Foxp3 (FOXP3 in humans) and can express a number of surface markers that are key for their function, including CD25, cytotoxic T‐lymphocyte‐associated protein 4 (CTLA‐4), inducible T‐cell co‐stimulator (ICOS) and T‐cell immunoreceptor with Ig and ITIM domains (TIGIT).^8^ In both mouse and human studies, Tregs are often defined as CD3^+ ^CD4^+ ^CD25^+ ^Foxp3^+^ cells, although Treg cells comprise a diverse population. During development, the first set of Tregs are formed in the thymus, making up the natural Treg population (nTreg), while a second type of Tregs can be induced in the periphery from naïve CD4 T‐cells [induced Tregs (iTreg)/peripheral Tregs (pTreg)] in the presence of specific cytokines such as interleukin (IL)‐2 and transforming growth factor beta (TGF‐β); however, the expression of Foxp3, and indeed regulatory activity, is variable depending on the level of demethylation at the *Foxp3* locus, and recent studies have identified a number of Treg subtypes delineated by CD25, Foxp3 and the epigenome.^9^ Regulatory activity can also be found in other lymphocyte subsets, such as the Foxp3^−^IL‐10^+^ Tr1 cells (previously defined as Th3^10^), although their role in controlling immune responses in helminth infection has yet to be established.

Regulatory T‐cells are critical in the prevention of autoimmunity and other forms of immune dysregulation, therefore these cells are likely to allow the parasite to not only survive for longer but also protect the host from a potentially pathogenic immune response. Hence, a large proportion of helminth‐infected individuals do not mount an inflammatory response to the parasite, which otherwise would cause ‘collateral damage’ in the infected tissues.

## Activity of Tregs in human helminth infection

In humans, strong links have frequently been identified between helminth infection and Treg cell activity, particularly in individuals who are asymptomatic or hypo‐responsive during infection (Table 1). It is important to note that in human studies, unlike in laboratory models, co‐infection with other pathogens is common, and there is great variability in the frequency and intensity of exposure to infection. Despite these confounding factors, however, some clear relationships have emerged.

**Table 1:** Human helminth Treg associations

Human disease [pathogen(s)]	Evidence for Tregs	Treg type/markers used	Reference
Ascariasis (*Ascaris lumbricoides*)	Blood samples from infected individuals had higher Treg numbers compared with uninfected controls	CD4^+^ CD25^+^	129
Hookworm infection (*Necator americanus*)	Higher levels of circulating Tregs compared with healthy non‐infected donors	CD4^+ ^CD25^+ ^FOXP3^+^ also expressed CTLA‐4, GITR, IL‐10, TGF‐β and IL‐17	130
Lymphatic filariasis (*Wuchereria bancrofti*)(*Wuchereria bancrofti* and *Mansonella perstans*)	Patients with lymphoedema had lower Treg levels compared with asymptomatically infected individuals	PBMCs measured for FOXP3, GITR, TGF‐β and CTLA‐4 by RT‐PCR	14
	Infected individuals had significantly increased frequencies of aTreg/Tr1 and nTreg compared with healthy controls	aTreg/Tr1 were CD4^+ ^IL‐10^+^ FOXP3^−^ nTregs were CD4^+ ^CD25^+ ^FOXP3^+^CD127^−^	12
(*Brugia timori*)	Asymptomatic microfilaraemics showed stronger Treg activity	CD4^+^CD25^(hi)^ cells depleted *in vitro*	13
Onchocerciasis (*Onchocerca volvulus*)	Hyper‐reactive onchocerciasis patients have deficiency in Tregs	CD4^−^ FOXP3^+^ CD25^(hi)^	15
Schistosomiasis (*Schistosoma mansoni*)	Elevated FOXP3‐expressing Tregs, expression of activation markers, anti‐helminth treatment Treg numbers return to baseline	CD3^+ ^CD4^+ ^CD25^+^	11
(*Schistosoma haematobium*)	Increased active Treg frequency, after anti‐schistosome treatment Treg numbers decrease but suppressive capacity remains	CD4^+ ^CD25^hi ^FOXP3^+^	131
Strongyloidiasis (*Strongyloides stercoralis*)	Tregs increased in patients with HTLV‐1 co‐infection compared with *Strongyloides* infection alone	CD4^+ ^CD25^+ ^FOXP3^+^ in PBMC	132
	Treg numbers increased in the duodenum of co‐infected patients compared with healthy controls	FOXP3 expression by IHC	133
Taeniasis/Cysticercosis (*Taenia solium*)	Significantly increased frequency of Tregs in patients with neurocysticercosis compared with healthy controls	CD4^+ ^CD25^high^ FOXP3^+^(Treg) CD4^+ ^CD25^high ^IL‐10^+^ (Tr1)	134

aTreg/Tr1, adaptive Treg; CTLA‐4, cytotoxic T‐lymphocyte‐associated; GITR, glucocorticoid‐induced tumour necrosis factor receptor‐related protein; HTLV‐1, human T‐cell lymphotropic virus type 1; IHC, immunohistochemistry; nTreg, natural Treg; PBMC, peripheral blood mononuclear cell; RT‐PCR, reverse transcription polymerase chain reaction.

In the case of *Schistosoma mansoni,* infection is associated with elevated numbers of FOXP3‐expressing Tregs, and these cells are also more active during helminth infection as indicated by expression of programmed cell death protein 1 (PD‐1) and CD45RO. However, after clearance with the anti‐schistosomal drug Praziquantel, this population returns to baseline.^11^ An increase in nTregs in addition to expanded IL‐10 producing Tr1 cells and Th17 cells has been shown in filarial infected individuals in comparison to uninfected controls.^12^ During filarial infections, peripheral T‐cells are typically unresponsive to parasite antigen, but responses could be rescued *in vitro* by depletion of CD25^(high)^ Tregs.^13^ There is also evidence to suggest that Tregs are important in preventing parasite‐associated pathologies. In patients infected with *Wuchereria bancrofti,* the major causative agent of lymphatic filariasis, those with lymphoedema have significantly enhanced Th1 and Th17 responses and lower Treg levels in comparison to asymptomatically infected individuals,^14^ while in hyper‐reactive onchocerciasis (river blindness) there is a deficiency in FOXP3^+ ^CD25^(high)^ Tregs.^15^ A recent study on rural Indonesians infected with soil‐transmitted helminths showed that CTLA‐4 and CD38, HLA‐DR, ICOS or CD161 co‐expressing Tregs were expanded compared with both urban‐dwelling Europeans, and urban‐residing Indonesians, excluding ethnicity as a major factor for this difference.^16^

Interestingly, both FOXP3^−^IL‐10^+^ Tr1 and FOXP3^+^ Tregs are associated with an isotype switch from IgE to IgG4 *in vitro* and during helminth infection.^17^ IgG4_,_ which does not exist in the mouse, is a strongly anti‐inflammatory isotype as it interacts poorly with cell‐bound Fc receptors, and also is functionally monovalent due to interchange between heavy‐light chain half‐molecules following secretion by B‐cells.^18^ Notably, IgG4 is promoted by IL‐10 and competes for the same epitopes as the strongly anti‐parasitic IgE isotype, thus suppressing IgE‐dependent allergic responses. During helminth infection, individuals have high levels of both IgG4 and IgE,^19^ with greater IgG4 : IgE ratios in asymptomatic infections with high Treg activity. These studies are supported by treatment with anthelmintic drugs, which rapidly reduce circulating IgG4 levels indicating another mechanism by which Tregs are involved in immunosuppression by helminths.^20^, ^21^

At a broader level, poor BCG vaccine responses have been found in helminth‐infected individuals, as BCG vaccinees show suppressed inflammatory cytokine profiles to purified protein derivative (PPD/tuberculin) antigen and strong TGF‐β production, both of which are reversed by anthelmintic treatment.^22^ Tregs are implicated in the poor BCG vaccine response, as evidenced by the reduced *in vitro* T‐cell proliferative responses to both BCG and malaria, and is recovered once Tregs are removed from these cultures.^23^ A more recent study on tuberculosis‐infected migrants in the UK showed that those who were co‐infected with helminths had a higher Treg frequency and lower interferon (IFN)‐γ^+^ CD4 T‐cells than those without a helminth infection, and furthermore that anthelmintic treatment reversed this effect.^24^

Overall this body of evidence supports a central role for parasite‐induced Tregs in immune regulation by helminths. Understanding the mechanisms by which this induction occurs may provide essential knowledge for anti‐helminth vaccinations and drug treatments.

## Mouse models of infection – expansion and manipulation of Tregs

Many mouse helminth models also show an expansion in Tregs, both from activating the nTreg population as well as *de novo* induction of pTregs, with significant increases by 3–7 days post‐infection.^25^, ^26^, ^27^, ^28^, ^29^ For example, both *Litomosoides sigmodontis* and *Heligmosomoides polygyrus* infections drive an early Treg expansion that is primarily made up of nTregs.^25^, ^28^ In addition, induction of early Foxp3^+^ cells by the filarial nematode *Brugia malayi* requires live, rather than heat‐killed, parasites.^26^ In conjunction with a quantitative expansion of host Tregs, helminths also induce expression of activation markers and cytokines including CD103, CTLA‐4 and TGF‐β, indicating that not only are Tregs induced but they may also express a more suppressive phenotype.^25^, ^27^

Helminth‐induced Tregs are essential for long‐term parasite survival within the immunocompetent host as removal of Tregs can result in clearance of the infection, whereas expansion of the Treg compartment with IL‐2 renders the host more susceptible.^30^ The strength of effect depends on the specific mouse model of helminth infection and the different depletion/induction methods employed, as summarized in Table 2. One approach has been to use antibody depletion of CD25^+^ cells (clone PC61), which removed most but not all Tregs. As CD25 is also expressed on activated effector cells, the anti‐CD25 is given before infection; Treg depletion in this manner increased host Th2 cytokine responses and enhanced parasite killing.^31^, ^32^, ^33^, ^34^, ^35^, ^36^ Additional anti‐CTLA‐4 (clone UC10‐4F10‐11) or anti‐glucocorticoid‐induced tumour necrosis factor receptor (GITR) (DTA‐1) inhibition alongside anti‐CD25 showed a further enhanced worm killing and elevation of Th2 cytokine responses, which may be attributed to ‘reawakening’ the effector T‐cell population in the absence of Treg influence.^32^, ^37^, ^38^

**Table 2:** Mouse parasite Treg interventions

Parasite	Mouse strain	Treatment	Role of Tregs	Reference
*Brugia pahangi*	BALB/c	CD25 depletion	Depleting CD25‐expressing cells increased Ag‐specific Th2 responses	31
*Heligmosomoides polygyrus*	C57BL/6	DEREG	Early depletion of Tregs did not affect worm burden but Th2 responses were enhanced	27
	BALB/c	Recombinant IL‐2:anti‐IL2 complex	Worm persistence increased with increased Treg numbers	30
	BALB/c	Foxp3.LuciDTR on days 4, 6, 8 and 10	Increased CD4^+^ T‐cell activation and pathology	
	C57BL/6	Foxp3.LuciDTR on days 14−26 every 2 days	No change to parasite burden despite increased Th2 responses	
*Litomosoides sigmodontis*	BALB/c	Ab depletion – anti‐CD25 (PC61) and anti‐GITR	Dual depletion results in increased killing of the parasites and enhances IL‐4, IL‐5 and IL‐10 responses	32
	BALB/c	Ab depletion – anti‐CD25 (PC61) and anti‐CTLA4 (UC10‐4F10‐11)	Dual depletion results in enhanced parasite killing, cytokine responses unaffected	135
*Schistosoma japonicum*	BALB/c	Ab depletion – anti‐CD25	Reduced Treg numbers were associated with reduced worm burden and increased IFN‐γ	33
	BALB/c	Ab depletion – anti‐CD25 and anti‐CTLA‐4	Dual depletion results in enhanced parasite killing	37
*Schistosoma mansoni*	C57BL/6	Ab depletion – anti‐CD25 (PC61) on day −3 and day 35	Enhanced egg destruction, increased pathology	34
	C57BL/6	Ab depletion – anti‐CD25 (PC61)	Tregs control Th2 colonic granulomas and reduce pathology during infection	136
*Strongyloides ratti*	BALB/c	DEREG mice	Early depletion of Tregs improved worm expulsion and Th2 responses	29
	BALB/c	CTLA‐4 blockade	Moderately increased Th2 and decreased Th1, limited enhancement to worm expulsion	
	BALB/c and C57BL/6	DEREG mice	Early depletion of Tregs improved worm expulsion in BALB/c mice but not C57BL/6 due to low IL‐9 production in this strain	40
*Trichinella spiralis*	C57BL/10	Ab depletion with PC61	Enhanced Th2 cytokine response to parasite Ag, no significance on larval burden	35
*Trichuris muris*	C57BL/6	Ab depletion – PC61 (anti‐CD25) and anti‐GITR	Treg depletion results in increased gut pathology but only anti‐GITR results in earlier worm expulsion	36
	C57BL/6	DEREG mice	Early Treg depletion enhances ability to clear the parasite, late Treg depletion reduced worm clearance. Both treatments resulted in enhanced Th2 responses	41

Ab, antibody; Ag, antigen; CTLA‐4, cytotoxic T‐lymphocyte‐associated protein 4; DEREG, ‘depletion of regulatory T‐cell’ mouse strain using diphtheria toxin‐induced ablation of Foxp3^+^ cells; Foxp3.LuciDTR, express knocked‐in diphtheria toxin receptor, although a different line function similar to DEREG mice; GITR, glucocorticoid‐induced tumour necrosis factor receptor‐related protein; IFN‐γ, interferon gamma; Treg, regulatory T cell.

A second approach has been to fully deplete Tregs in transgenic mice expressing the diphtheria toxin receptor (DTR) under the Foxp3 promoter. Toxin administration to these mice showed that Treg depletion during the early stages of infection may induce protective immunity; however, depletion at the later stages shows enhanced host pathology and lack of worm expulsion.^29^, ^30^, ^39^ The differing results between antibody depletion and DTR mice may be explained by the residual Treg population in the former scenario, and indicate that a basal level of Tregs may be essential for controlling host pathology and permitting a coherent Th2 response to develop in the face of, for example, IFN‐γ responses against commensal bacteria. Furthermore, complete Treg depletion in DTR mice is only possible for short periods of time due to increased mortality, rendering it difficult to determine the role of Tregs during the full time course of a helminth infection.

As summarized in Table 2, in helminth models such as *H. polygyrus*, *L. sigmodontis*, *Schistosoma japonicum* and *Strongyloides ratti*, Tregs have been shown experimentally to be important for parasite survival; however, this is not the case for other species such as *Trichuris muris*, which dramatically reduces the proportion of Tregs during infection and Treg depletion in this model has no effect on worm survival.^42^, ^43^, ^44^ Clearly, helminths have adopted multiple strategies to evade host immunity, and therefore not all species are reliant on the induction of Tregs for survival within the host, and Treg‐directed mechanisms may be more or less important according to the genotype of the host.^40^, ^45^

## Pathways by which helminths induce Tregs: directly and indirectly

**Figure 1:**
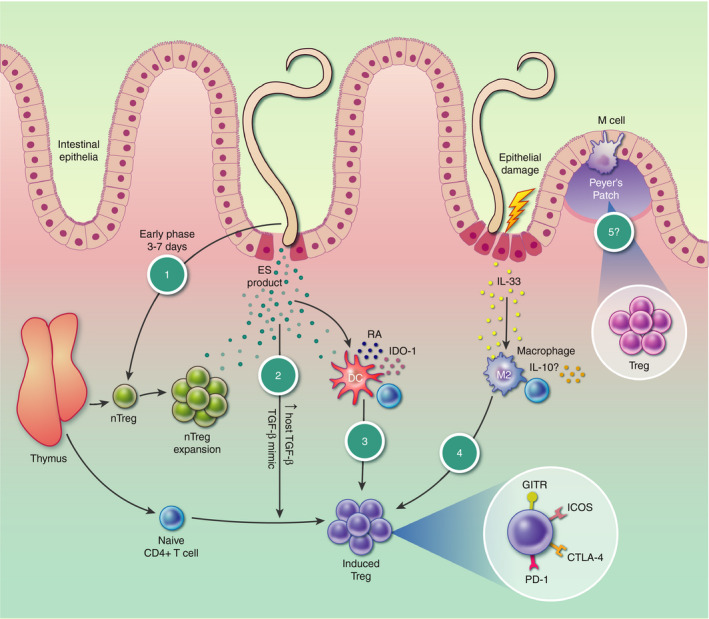
Overview of events in Treg expansion by helminths. (1) Early expansion of natural regulatory T‐cells (nTreg) by helminths within the first 3–7 days. (2) Helminth excretory−secretory (ES) products induce naïve CD4^+^ T‐cells to become Tregs. (3) ES products polarize dendritic cells (DCs) towards a tolerogenic phenotype capable of inducing Tregs. (4) M2 macrophage polarization following IL‐33 release by damaged epithelium, which then induce Tregs through an undefined mechanism. (5) Helminth proximity to Peyer's patch (PP) causes Treg expansion. It is as yet unclear whether Treg events (1)−(4) occur in either or both the lamina propria of the small intestine and the draining mesenteric lymph nodes, and hence no distinction is made in this figure.

Upon infection, many helminths drive an expansion or recruitment of nTregs during the early stages as indicated by Helios^+^ staining,^30^, ^46^ while nTreg depletion using anti‐CD25^+^ antibody prior to infection shows that these cells have a role in parasite persistence.^28^ Treg expansion in *H. polygyrus*‐infected mice is greatest in the Peyer's patches (PP) that are in contact with the parasite, indicating that secreted products may act in a local manner to promote Treg expansion.^47^ While it is not known what specifically drives the expansion of nTregs, there have been many studies looking at the factors that increase overall Treg numbers during helminth infection indicating specific pathways by which iTreg/Tr1 cells are induced,^48^ and these are summarized in Fig. 1.

Many helminths secrete a plethora of proteins that are able to interact with the host, in the case of *H. polygyrus* several hundred proteins have been identified in the excretory‐secretory products (HES)^49^ and, furthermore, these products are able to induce Tregs from naïve CD4^+^ T‐cells *in vitro*.^50^ Further analysis identified the active protein, named *Hp‐*TGM [*H. polygyrus* TGM (TGF‐β mimic)], which mimics the function of TGF‐β by binding to the TGF‐β receptors and inducing Smad signalling.^51^ Although *Hp‐*TGM has no structural homology with mammalian TGF‐β, it is a potent inducer of mouse and human Foxp3^+^ Treg cells *in vitro.* Elaboration of a factor that drives Treg induction argues that their expansion during helminth infection is not merely a host homeostatic mechanism to rein in overstimulation but an adaptive evolutionary strategy on the part of the parasite to maximize their survival in an immune environment.

Interestingly, *Hp‐*TGM is part of a larger gene family made up of 10 members based on a similar sequence, with multiple variants able to induce Tregs.^52^ This redundancy indicates that TGF‐β mimicry is a central survival ploy by this parasite. However, other helminths appear to depend upon host TGF‐β to indirectly induce Tregs. For example, in *S. mansoni* infection, soluble egg antigens (SEA) are able to upregulate host cell TGF‐β secretions and induce Foxp3^+^ Tregs in a TLR2‐dependent manner.^34^, ^53^

Further examples substantiate that helminths and their products act on bystander cell types, which are then able to induce Tregs in an indirect manner. One such hypothesized mechanism is through the induction of tolerogenic dendritic cells (DCs) that express a range of immunosuppressive factors, including IL‐10, indolamine 2,3‐dioxygenase (IDO), programmed death‐ligand 1 (PD‐L1), retinoic acid (RA) and TGF‐β, which are known to drive naïve T‐cells towards a Treg phenotype.^54^, ^55^ This pathway has been implicated in *Trichinella spiralis* infection in which DCs exposed to muscle larvae excretory/secretory (ES) product are able to expand IL‐10 and TGF‐β‐producing Foxp3^+^ Tregs in an IDO‐1‐dependent manner.^56^ Bone marrow‐derived DCs treated with *H. polygyrus* ES also have reduced co‐stimulatory molecule and cytokine expression, and these cells are able to induce IL‐10‐secreting T‐cells (Tr1, regulatory cells) capable of suppressing effector T‐cell responses.^57^

An emerging area of interest for Treg induction *in vivo* relates to the role of alarmins and tissue‐derived cytokines in promoting the regulatory environment. In addition to factors secreted by the parasites, the epithelial damage caused by helminths results in release of alarmins such as TSLP and IL‐33,^58^, ^59^ which may also be involved, indirectly or directly, in expanding Treg numbers during infection. For example, macrophages express ST2, a receptor for IL‐33, and in the presence of this cytokine can be polarized to an M2 phenotype leading to IL‐10 upregulation and consequent Treg expansion.^60^

## Treg locations: subtypes and surface markers

Helminths are able to infect a range of tissues outside of the gut, including the lung, pleural cavity, peritoneal cavity and liver.^26^, ^38^, ^46^, ^61^ In each of these different tissue sites, the immune environment these helminths encounter will be unique, with tissue‐specific immunological properties including Treg features.^67^, ^68^ While our understanding of helminth−Treg interactions in the tissues is still limited, there are certainly conserved markers of Treg activation that are induced in multiple helminth‐tissue settings.

**Figure 2:**
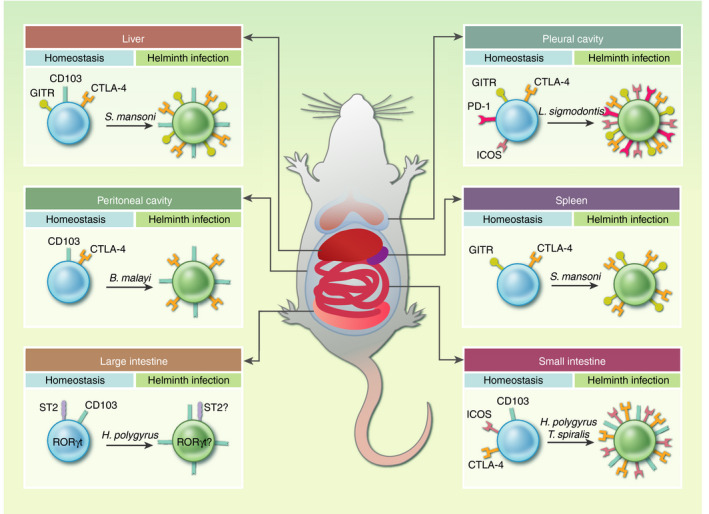
Tregs in response to helminth infection in different tissues. Liver, upregulation of GITR, CD103 and CTLA‐4 on Tregs in response to *Schistosoma mansoni* infection and exposure to eggs trapped in the liver; peritoneal cavity, CD103 and CTLA‐4 on Tregs are upregulated when infected with the filarial nematode *Brugia malayi*; large intestine, increase in CD103 expression on Tregs in mice carrying duodenal infection with *Heligmosomoides polygyrus*, Treg expression of RORγt and ST2 are as yet unknown during helminth infection; pleural cavity, *Litomosoides sigmodontis* infection upregulates ICOS, GITR, PD‐1 and CTLA‐4 expression on Tregs; spleen, *S. mansoni* infection increases the expression of GITR and CTLA‐4 on Tregs; small intestine, *Trichinella spiralis* infection induces high levels of CTLA‐4 expression on Tregs and *H. polygyrus* infection upregulates CD103 and CTLA‐4 as measured on Tregs in the mesenteric lymph nodes, a surrogate of the populations in the small intestine lamina propria. CTLA‐4, cytotoxic T‐lymphocyte‐associated protein 4; GITR, glucocorticoid‐induced tumour necrosis factor receptor; ICOS, inducible T‐cell co‐stimulator; PD‐1, programmed cell death protein 1; RORγt RAR‐related orphan receptor gamma.

Expression of CD103, a beta integrin associated with tissue residency, is shown to be upregulated on Tregs in the liver,^63^, ^64^ peritoneal cavity,^26^ large intestine^61^ and small intestine^25^ during helminth infection compared with steady‐state. Upregulation of CD103 on Tregs may be a conserved mechanism seen in *B. malayi*, *H. polygyrus* and *S. mansoni* infection (Fig. 2), indicating that retention of Tregs in the tissues is important for their function during helminth infections.^25^, ^26^, ^61^, ^63^, ^64^

Co‐inhibitory molecules are commonly upregulated by helminth infection. CTLA‐4 (also referred to as CD152) is upregulated on Tregs in many infections, including *B. malayi*, *L. sigmodontis*, *S. mansoni* and *T. spiralis*.^26^, ^38^, ^62^, ^65^ When CTLA‐4, a member of the CD28 family, binds to CD80 or CD86 on DCs they become tolerogenic, and thus downstream T‐cell responses are inhibited. Interestingly, the checkpoint inhibitor GITR is also upregulated during helminth infection. GITR functions as a Treg inhibitor when bound to its ligand, GITRL, which allows the activation and expansion of T effector cells. It has been shown that stimulating GITR and thus decreasing Treg responses during *L. sigmodontis* infection causes increased Th2 numbers and cytokine output.^66^ GITR is also upregulated during *S. mansoni* and *L. sigmodontis* infection.^38^, ^62^ Furthermore, other co‐inhibitory molecules can also be found on the surface of Tregs responding to helminth infection including PD‐1 and ICOS, which indicate that not only are Tregs expanded during helminth infection but are also potentially more actively suppressive.^38^, ^46^, ^62^

An emerging paradigm has been that Treg co‐expression of transcription factors previously associated with Th effector subsets enables them to co‐migrate to the same sites and thereby fulfil their suppressive action. For example, RAR‐related orphan receptor gamma (RORγt), a transcription factor that typically defines Th17 cells, is expressed on a group of Tregs found in the large intestine.^69^ A recent study has indicated that this unusual subtype is also involved in the regulation of Th2 responses, an idea that is supported by RORγt^fl/fl^Foxp3^cre^ mice, which were able to expel *H. polygyrus* more efficiently than C57BL/6 counterparts.^69^

As discussed above, innate alarmins such as IL‐33 have a major impact on the T‐cell population. Hence, the report of ST2 (IL‐33R) expressing Tregs in the colon is particularly interesting.^70^ However, ST2‐deficient mice have normal Treg responses to *H. polygyrus* infection and indeed are more susceptible to the parasite,^71^ highlighting the contrasting role of IL‐33 in different tissues and settings. Certainly, more investigation is required to study the dynamics of these newly identified subtypes during helminth infection, and in particular to compare and contrast the phenotypes of Tregs induced by helminths migrating through, or establishing in, different tissues such as the skin, lungs, vasculature and intestinal tract.

## Therapeutic potential – epidemiological evidence in humans

As discussed above, regulatory T‐cells may protect the host against the excessive immunopathological responses to helminth infection, and maintain an immunological compromise that benefits host health while tolerating some degree of parasite infestation. The promotion of regulatory T‐cells by helminths may have further, albeit less expected, beneficial effects for the host by downregulating responsiveness to other coinciding antigens, and therefore reducing the impact of allergens, autoantigens and other infectious agents. This is one strand of the postulated ‘hygiene hypothesis’, which would be consistent with experimental and epidemiological evidence gathered from both mouse and human studies.^72^, ^73^

Several studies have reported that helminth‐infected individuals have a lower allergic response to allergens, such as the house dust mite (HDM), as is the case for children infected with *Schistosoma haematobium* ^74^, ^75^ and *S. mansoni*.^76^, ^77^ This effect is further supported by an enhancement of skin test reactivity in children following anthelmintic treatment, providing evidence of causality between helminth infection and reduced allergy,^78^, ^79^ Studies in which pregnant mothers were treated with anthelmintics, in the hopes that parasite elimination would reduce maternal anaemia and enhance fetal growth, showed that there was actually a significant adverse effect with increased rates of infantile eczema.^80^, ^81^ It is important to note that particular helminth species can be more strongly linked to a protection against allergy, such is the case for hookworm, and that the intensity of the infection may also be key to the protective effects. Together these studies, and others, built a body of evidence that helminths exert a protective effect against allergies and provide an environmental explanation for higher levels of eczema and asthma in the developed world.^82^, ^83^, ^84^

While there has been a parallel increase in the incidence of autoimmune diseases in developed countries, there is sparse epidemiological evidence for parasite immunosuppression reducing autoimmunity. Levels of auto‐reactive anti‐nuclear antibodies (ANA), which are central for the diagnosis and classification of autoimmune diseases, were found to be lower in individuals infected with *S. haematobium* compared with age‐matched uninfected cohabitants. Moreover, 6 months after anti‐helminthic treatment, levels of ANA significantly increased, implying that the parasite is able to generate conditions in which autoimmunity is suppressed.^85^ The most striking indication of Treg involvement in helminth protection against autoimmunity was determined in a multiple sclerosis (MS) patient cohort in Argentina, in which MS patients unintentionally acquired gastrointestinal helminth infection with a variety of species. Those infected had increased TGF‐β and IL‐10 levels, as well as elevated Treg and Breg activity. The infected patients also showed significantly lower numbers of disease exacerbations and fewer magnetic resonance imaging (MRI) lesion changes in comparison to uninfected MS patients over the same period of time.^86^ A small number of these patients were subsequently given anti‐parasitic treatment, which leads to an increase in clinical and radiological disease. This increase in MS severity was also associated with a reduction in TGF‐β‐ and IL‐10‐secreting cells and reduced FOXP3^+^ Tregs within 3 months post‐anthelmintic treatment.^87^

Although we have highlighted particular studies indicating that helminth infection may protect against inflammatory diseases, there are also instances in which worms may exacerbate disease. In a mouse model setting, *H. polygyrus* promotes colon cancer following DSS‐driven inflammation^61^ and enhances colitis provoked by *Citrobacter* infection.^88^ In humans, anthelmintic treatment of individuals in an area highly prevalent for *Ascaris* resulted in an overall improvement of asthma within the population.^89^ This further highlights the double‐edged nature of live parasites and the benefits of identifying individual molecular products that induce Tregs, which can then be used as a therapeutic treatment for inflammatory diseases in the future.

### Evidence for a role of helminth Tregs in suppression of inflammatory disease

To clarify the role Tregs may be playing in the helminth‐induced bystander immunosuppression, we look to mouse models of disease. Helminths have been used in a range of autoimmune or allergy models, showing that parasites are able to successfully suppress inflammatory diseases, as recently summarized by fellow colleagues^90^, ^91^ and ourselves.^92^ These studies have determined that Tregs are an important cell type mediating helminth protection, although in some models helminth protection is Treg‐, or even T‐cell‐independent, indicating that the mechanism of protection may be parasite and disease model specific.

In *H. polygyrus* a role for Tregs have been demonstrated for immune suppression in a range of different disease models, including allergic airway inflammation with HDM, ovalbumin (OVA)‐specific airway allergy, type 1 diabetes in non‐obese diabetic (NOD) mice and inflammatory bowel disease.^93^, ^94^, ^95^ Similarly, during *S. mansoni* infection there is evidence to suggest Tregs play a role in suppression of OVA‐specific airway inflammation, and in some studies protection is both T‐cell and IL‐10‐dependent, indicating that Tr1 cells may also be important immune regulators during helminth infection.^84^, ^96^, ^97^

## Human helminth therapy trials – past, present and future?

Deliberate infection of humans with helminths to dampen inflammatory disorders has been mooted for several decades, ever since a report that self‐infection with *Necator americanus* hookworms abolished hay fever.^98^ Such anecdotal reports continue, with for example a colitis sufferer showing benefit from self‐treating with the human whipworm *Trichuris trichiura*.^99^ More controlled trials of live helminth therapy started in 2006 using the pig whipworm, *Trichuris suis*, which establishes a transient infection in humans, and potentially less pathogenic than human‐infective helminth species; furthermore, *T. suis* ova (TSO) administration successfully treated a macaque monkey colony suffering idiopathic bowel dysfunction.^100^ Notably, in neither of these studies did *Trichuris* spp. infection raise expression of FOXP3^+^ Tregs, which were generally found to be more frequent in inflammation than in healthy tissues and controls.

Early, small‐scale trials of TSO in Crohn's disease and ulcerative colitis provided promising results,^101^, ^102^ followed by larger clinical trials not only for IBD, but also rhinitis and MS, which have recently been reviewed by a number of authors;^103^, ^104^, ^105^, ^106^, ^107^, ^108^ helminth therapy has even extended to autism.^109^ The outcomes for inflammatory bowel disease (IBD) and MS remain inconclusive,^106^, ^110^, ^111^ with modest effects at best,^112^ and generally falling short of statistical significance. However, a significant benefit was found for hookworm treatment of patients with Coeliac disease, many of whom regained gluten tolerance, accompanied by a significant increase in FOXP3^+^ Tregs among the intraepithelial lymphocytes,^113^ although not in peripheral blood.^114^

Several commentators have identified reasons why helminth therapy has been less efficacious than may have been hoped, focussing on whether early‐age, longer‐term and/or higher‐intensity infections may be required,^105^ whether a human parasite is more appropriate than a porcine one,^104^ if the entirely enteric location of whipworms lacks a systemic presence and, perhaps most uncertain, whether the human immune system varies significantly in responses to helminth infection.^115^ As yet, there appears to be no common immunoregulatory pathway invoked by experimental human helminth infection, with the paucity of evidence for Treg involvement being perhaps the most surprising.

Overall, helminth therapy for human inflammatory disorders holds some important lessons, and some caveats, for the future. Live infection remains an imperfect art, with parasite biology dictating dose, longevity and location of the therapeutic agents; these agents, presumed to be secreted products, are themselves poorly defined as are the host targets of parasite immune modulation. Hence, while recent work has provided proof‐of‐concept that helminths may offer innovative anti‐inflammatory treatments, further advances now require identification of helminth modulatory molecules and their modes of action, so that rational and defined new pharmaceutics can be developed for therapy.

## Moving forward into the molecular era

A number of exciting parasite proteins have recently been identified as potential immune modulators for inflammatory disease;^48^ however, for the purpose of this review we will focus on molecules that exert their effects through Tregs, either directly or indirectly. HES are capable of directly inducing Tregs *in vitro* that effectively suppress both *in vitro* effector cell proliferation as well as *in vivo* allergic airway inflammation.^50^ As mentioned earlier, the component within HES has been identified as *Hp*‐TGM and signals through TGF‐β receptors to induce Foxp3^+^ Tregs *in vitro*. In a model of allograft rejection, mice receiving *Hp*‐TGM or HES had an extended median survival with reduced inflammation and elevated Foxp3^+^ Treg numbers in the allograft draining lymph nodes compared with controls.^51^ Interestingly, manipulation of the TGF‐β pathway is not unique to *H. polygyrus*, with active ligands identified from other helminth species.^48^, ^90^, ^116^

While examples of helminth molecules directly driving Treg differentiation remain few, a greater range of helminths appear to induce Tregs indirectly. The proteome analysis of the hookworm *Anyclostoma caninum* revealed two proteins named anti‐inflammatory protein (AIP)‐1 and AIP‐2 that were subsequently identified as having immunosuppressive properties. Intraperitoneal administration of AIP‐1 was shown to limit inflammatory cell infiltrate, increased IL‐10 and TGF‐β production, and recruited Tregs to the site of inflammation in a mouse model of colitis.^117^ On the other hand, AIP‐2 was effective at suppressing inflammation and pathology in a mouse model of asthma, and expansion of CD11c^+^ DCs and Foxp3^+^ Tregs were essential for this disease protection.^118^ The study by Navarro *et al.* also showed that AIP‐2 suppressed the proliferation of T‐cells from patients with HDM allergy *ex vivo* indicating that this protein might be a potential therapeutic for allergic asthma in humans.

Similarly, *S. mansoni* SEA are capable of protecting NOD mice against the development of type‐1 diabetes in a Treg‐dependent manner. This was indicated as splenocytes from SEA‐treated mice were unable to transfer diabetes, whereas splenocytes that were CD25^+^ T‐cell depleted had a restored ability to transfer diabetes.^53^ The SEA contains a well‐characterized glycoprotein, ω‐1, that is able to drive Foxp3^+^ Treg numbers, and NOD mice immunized with ω‐1 were also protected from diabetes. It has been identified that ω‐1 induces Foxp3 expression in NOD mouse CD4^+^ T‐cells through the induction of tolerogenic DCs that produce TGF‐β and retinoic acid.^55^

Many helminths secrete cysteine protease inhibitors (CPIs) with immunomodulatory properties.^119^, ^120^ A recent study on recombinant *Ascaris lumbricoides* rA1‐CPI in a mouse model of allergic airway inflammation with HDM showed that rA1‐CPI reduced airway inflammation and hyper‐reactivity. A significant reduction in Th2 cytokines and increased Tregs in spleen, as well as increased IL‐10 levels in the bronchoalveolar lavage and splenocyte cultures in rA1‐CPI‐treated mice compared with controls. Furthermore, this effect was partially blocked by anti‐IL‐10‐receptor suggesting the disease suppression seen is in part mediated through IL‐10 signalling.^121^

These parasite‐derived proteins could be used to induce Foxp3 expression *in vivo,* making them excellent candidates for use in clinical trials on inflammatory diseases in which Treg numbers are reduced or dysfunctional. Furthermore, molecules such as *Hp*‐TGM have no structural homology to mammalian TGF‐β therefore, unlike native TGF‐β, may retain biological activity for a long period of time. Although the use of parasite molecules in place of the helminth itself may prove to be a fruitful venture for clinical therapy, the induction or expansion of Foxp3^+^ Tregs by parasite molecules *in vitro* and subsequent transplantation into diseased patients may be another path worth exploring.

## Will trials with helminth‐induced Treg transfer be effective?

Given that clinical trials with live parasites have shown very little consistency in inflammatory disease thus far, future studies may be directed towards the use of helminth products for Treg transfer clinical trials. Animal models have identified that adoptive transfer of Tregs from *S. mansoni*‐ or *H. polygyrus*‐infected mice can protect against inflammatory disease, suggesting these cells can exert systemic down‐modulation of bystander immunopathologies.^50^, ^53^, ^93^ Moreover, a major challenge in Treg therapy is to direct cells to the site of inflammation, and that they mediate the appropriate suppressive mechanism required to treat the disease in question.^122^ Given that some helminth products induce RA, which is important for the upregulation of gut‐homing receptors such as CCR9 and integrin‐α4β7, this may be advantageous for Treg therapeutics in IBD in trafficking cells to the site of inflammation.^55^, ^123^ Additionally, as discussed earlier in the review, Tregs during helminth infection express high levels of CTLA‐4, PD‐1 and ICOS on their surface, indicating that these cells potentially represent a more active Treg population that would be efficient suppressors of inflammatory diseases.

Currently, there are several concerns about the efficacy and risks of Treg therapy in humans. An overactive Treg compartment might compromise immunity to other infections, or even permit outgrowth of tumours, in a reverse scenario to strategies targeting Tregs for cancer control.^124^ A more subtle concern for Treg therapy following *ex vivo* conversion and return to the same patient, is whether the injected Treg population will retain suppressor properties or if they will be converted into effector cells. Some studies have looked at the nTreg population switching to Th17 cells in the presence of IL‐6, rendering them a problematic population in autoimmune diseases such as rheumatoid arthritis and MS that are characterized by auto‐reactive Th17 cells.^125^ However, the pre‐treatment of these cells with IL‐2, TGF‐β and/or RA make these cells resistant to effector cell conversion and allows them to retain suppressive properties.^126^, ^127^ This therefore identifies an area in which helminth products may be used to stabilize nTreg populations *ex vivo,* allowing them to remain potent immunosuppressors once transplanted.

To date, Treg transfer clinical trials have focused on *ex vivo* expansion of pre‐existing nTregs and implanting them back into patients,^128^ while very little work has been done on converting naïve T‐cells, or even pro‐inflammatory effector T‐cells, into Tregs before implantation. This could be a new area of research for helminth products. An advantage of inducing Tregs from naïve or effector CD4^+^ T‐cells is the generation of antigen‐specific Tregs, which may be directed toward the suppression of a specific autoimmune disease, and which may be absent from the pre‐existing nTreg population within the patient. Furthermore, as several inflammatory diseases are associated with a deficient number of nTregs, the ability to induce stable autologous Tregs from naïve CD4^+^ T‐cells would be an improvement on current Treg therapeutic options.

## Conclusion

The world of helminths has opened many new perspectives on immune regulation in general and Tregs in particular. Animal models have shown strong evidence that helminthic therapy can treat and/or prevent inflammatory diseases with some models dependent on Tregs; however, thus far the human clinical trials have been lacking in consistency or efficacy. Therefore we suggest that in the coming years the focus should be switched to individual helminth products as they provide a safer, more defined and directable therapeutic compared with live infection. Of particular interest are compounds that play a key role in the immunoregulatory network, either directly or indirectly inducing and expanding Tregs either *ex vivo* or *in vivo*, and with potential to stabilize and promote the function of this cell type as a future therapy across a broad range of chronic inflammatory conditions.

## Disclosures

The authors declare having no competing interests.
